# Decreased Serum Free Testosterone in Workers Exposed to High Levels of Di-*n*-butyl Phthalate (DBP) and Di-2-ethylhexyl Phthalate (DEHP): A Cross-Sectional Study in China

**DOI:** 10.1289/ehp.9016

**Published:** 2006-07-27

**Authors:** Guowei Pan, Tomoyuki Hanaoka, Mariko Yoshimura, Shujuan Zhang, Ping Wang, Hiromasa Tsukino, Koichi Inoue, Hiroyuki Nakazawa, Shoichiro Tsugane, Ken Takahashi

**Affiliations:** 1 Department of Environmental Epidemiology, Liaoning Provincial Center for Disease Prevention and Control, Shenyang, People’s Republic of China; 2 Epidemiology and Prevention Division, Research Center for Cancer Prevention and Screening, National Cancer Center, Tokyo, Japan; 3 Department of Environmental Epidemiology, University of Occupational and Environmental Health, Kitakyushu, Japan; 4 Department of Hygiene and Preventive Medicine, Showa University, School of Medicine, Tokyo, Japan; 5 Department of Infectious Disease, Shenyang Municipal Center for Disease Prevention and Control, Shenyang, People’s Republic of China; 6 Department of Analytical Chemistry, Faculty of Pharmaceutical Sciences, Hoshi University, Tokyo, Japan

**Keywords:** di-*n*-butyl phthalate (DBP), di-2-ethylhexyl phthalate (DEHP), free testosterone (fT), mono-*n*-butyl phthalate (MBP), mono-2-ethylhexyl phthalate (MEHP), occupational exposure

## Abstract

**Background:**

Observations of adverse developmental and reproductive effects in laboratory animals and wildlife have fueled increasing public concern regarding the potential for various chemicals to impair human fertility.

**Objective:**

Our objective in this study was to assess the effect of occupational exposure to high levels of phthalate esters on the balance of gonadotropin and gonadal hormones including luteinizing hormone, follicle-stimulating hormone, free testosterone (fT), and estradiol.

**Methods:**

We examined urine and blood samples of 74 male workers at a factory producing unfoamed polyvinyl chloride flooring exposed to di-*n*-butyl phthalate (DBP) and di-2-ethylhexyl phthalate (DEHP) and compared them with samples from 63 male workers from a construction company, group matched for age and smoking status.

**Results:**

Compared to the unexposed workers, the exposed workers had substantially and significantly elevated concentrations of mono-*n*-butyl phthalate (MBP; 644.3 vs. 129.6 μg/g creatinine, *p* < 0.001) and mono-2-ethylhexyl phthalate (MEHP; 565.7 vs. 5.7 μg/g creatinine, *p* < 0.001). fT was significantly lower (8.4 vs. 9.7 μg/g creatinine, *p* = 0.019) in exposed workers than in unexposed workers. fT was negatively correlated to MBP (*r* = −0.25, *p* = 0.03) and MEHP (*r* = −0.19, *p* = 0.095) in the exposed worker group. Regression analyses revealed that fT decreases significantly with increasing total phthalate ester score (the sum of quartiles of MBP and MEHP; *r* = −0.26, *p* = 0.002).

**Conclusion:**

We observed a modest and significant reduction of serum fT in workers with higher levels of urinary MBP and MEHP compared with unexposed workers.

Observations of adverse developmental and reproductive effects in laboratory animals and wildlife have fueled increasing public concern regarding the potential for various chemicals to impair human fertility. Included among the list of chemicals suspected of impairing human fertility are the phthalate esters (PEs), which are used extensively as plasticizers in household and consumer goods and in certain medical products. Each year 2–8 million tons of PEs are produced and consumed worldwide [Colborn et al. 1993; Toppari et al. 1996; World Health Organization (WHO) 1992]. Several studies have demonstrated the extent of exposure to PEs in the general population (Blount et al. 2000; Koch et al. 2003b; Silva et al. 2004b).

Phthalate monoesters, including mono-2-ethylhexyl phthalate (MEHP) and mono-*n*-butyl phthalate (MBP), are known testicular toxicants in rodents. The Leydig cells (LCs) and Sertoli cells (SCs) that play crucial roles in spermatogenesis and testosterone production are considered the primary targets of phthalate monoester toxicity (Akingbemi et al. 2004; Barlow et al. 2003; Foster et al. 2001; Gray et al. 2000; Jones et al. 1993; Mahood et al. 2005; Wang et al. 2005). Although adverse effects on male reproduction are suggested, research findings remain inconsistent (Kumar 2004). There is a large gap between results from studies investigating exposure to relatively high levels of PEs in a laboratory setting and the relatively low levels found in the general environment (Mylchreest et al. 2002). Both fetal and adult exposure to PE are suspected to contribute to impaired human fertility (Mahood et al. 2005; Parks et al. 2000; Skakkebaek et al. 2001).

Although many toxicologic studies on PEs have been conducted in the past decade, few epidemiologic studies have assessed the relationship between PE exposure and the effect on human reproduction. Obstacles that have hindered human studies include the long latency period from fetal exposure, low exposure levels, difficulties in sampling sperm, and the involvement of complex cell–cell interactions between the cells and hormones associated with the hypothalamo–pituitary–testis (HPT) system. Although several studies have demonstrated high levels of PEs in patients under dialysis or extra-corporeal membrane oxygenation and in workers having occupational exposures, very few studies have evaluated the effects of PE exposure on reproductive function (Cooper and Kavlock 1997; Duty et al. 2005; Hoppin 2003; Kavlock 1999; Main et al. 2006; Takahashi et al. 2004). Thus findings from epidemiologic studies are not conclusive as to whether exposure to environmental levels of PEs can cause sperm damage and/or disrupt the gonadal hormone balances in adult males.

Because occupational exposure to PEs is generally higher than environmental exposure, the workplace provides an appropriate setting to study the effects of PEs on reproductive function (Kumar 2004). The present study was designed to assess the effect of occupational exposure to high levels of di-*n*-butyl phthalate (DBP) and di-2-ethylhexyl phthalate (DEHP) on the circulating concentration and/or balance of free testosterone (fT), luteinizing hormone (LH), follicle-stimulating hormone (FSH), and estradiol (E_2_).

## Methods

### Subjects and sample collection

The study was conducted in a factory producing unfoamed polyvinyl chloride (PVC) flooring in Liaoning Province, China. DBP and/or DEHP were used as plasticizers in four similar production lines; the workers were exposed to DBP and/or DEHP by dermal contact and through dust inhalation. For our exposed workers group, we selected all 74 male workers currently working in the four production lines. The exposed workers were involved in raw material preparation and mixing (*n* = 14), filtering (*n* = 13), refining (*n* = 23), rolling and pressing (*n* = 14), packaging (*n* = 4), and other duties (*n* = 6). For a comparison group without occupational exposure to DBP and/or DEHP, we randomly selected 63 male workers from 89 employees of a construction company, group matched for age and smoking status. They were woodworkers (*n* = 24), bricklayers (*n* = 16), workers in material preparation and loading (*n* = 13), scaffolders (*n* = 4), reinforcing steel bar workers (*n* = 4), and electricians (*n* = 2).

Urine and blood samples were collected from each subject between 800 and 1100 hours on the same day, but not on the first day of the subject’s work week or the day after a night work shift. Peripheral blood was collected in an EDTA-2Na tube, and plasma was collected by centrifugation. A simple questionnaire was used to obtain lifestyle information, including smoking and alcohol consumption habits, personal plastic material usage, and consumption of soybean products. We developed a summary index for plastic material contact (SIP) to ascertain if the subject came into contact with plastic tableware (0/1), water or tea in polyethylene terephthalate bottles (0/1) and food packaging (0/1). The frequency of soybean product consumption (FSPC) was defined as the frequency per week of consuming soybean products.

This study was conducted in accordance with the Declaration of Helsinki (World Medical Assoiation 2004). All subjects volunteered to participate in the study and gave written informed consent.

### Chemicals, instruments, and analytical conditions for MBP and MEHP

Stock solutions of the standard chemicals were prepared in acetonitrile at a concentration of 100 mg/mL. We purchased MBP, MEHP, and d_4_-labeled internal standards from Hayashi Pure Chemical Industries (Osaka, Japan). Organic solvents for PE analysis and sample stock preparation were obtained from Kanto Chemical Co. (Tokyo, Japan). Ammonium acetate, acetic acid, and β-glucuronidase solution from *Escherichia coli* (85 U/mL) were purchased from Wako Pure Chemical Industries (Osaka, Japan).

Liquid chromatography-electrospray-ionization (ESI) tandem mass spectrometry (MS/MS) was performed using a Waters Alliance HT2795/Micromass Quattro micro system (Waters Co., Milford, MA, USA). The injection volume was 20 μL; the precolumn was a Mightysil RP-18 GP 5-2.0 (5 μm; Kanto Chemical Co.); and the analytical column was an Inertsil ODS-3 (2.1 × 50 mm, 5 μm; GL Sciences Inc., Tokyo, Japan). The column temperature was maintained at 40°C. The linear gradient program was as follows: 96%A/1%B/3%C (0 min), 4%A/1%B/95%C (3–7 min), and 96%A/1%B/3%C (7.2 min). We used a flow rate of 0.2 mL/min. For operation of the ESI-MS/MS, the flow rates of dry nitrogen for desolvation and cone gas were 350 and 50 L/hr, respectively, and the temperatures for desolvation and source were 350 and 100°C, respectively. The MS/MS data for MBP, MEHP, and the d_4_-internal standards were collected in negative ion mode by multiple reaction monitoring of the transition *m/z* 221 → 77 (MBP), *m/z* 277 → 134 (MEHP), *m/z* 225 → 81 (MBP-d_4_), and *m/z* 281 → 138 (MEHP-d_4_). The optimized parameters for ESI with monitoring ions of MBP and MEHP were a cone potential of −22 and −28 V and a collision energy of 17 and 16 eV, respectively. Other working conditions for the MS/MS in multiple reaction monitoring mode included a cone gas of nitrogen, a collision gas of argon, an interchannel delay of 0.1 sec, and repeats of one time span of 0.1 sec.

For cleanup and preconcentration determination, we modified a solid phase extraction (SPE) method for measuring urinary mono-phthalate ethers previously described by Blount et al. (2000) and Silva et al. (2004b). Briefly, a 3-mL aliquot from a human urine sample (spiked internal standard, 30 μL) was buffered with 100 mM ammonium acetate/acetic acid (1 mL, pH 6.2); after the addition of β-glucuronidase solution (50 mL, 4.25 U), the sample was sealed in a glass tube and gently mixed. This solution was then incubated at 37°C for 60 min to deconjugate the glucronidated phthalate metabolites. This reaction time was established in preliminary experiments, which showed maximal reaction at 60 min. We used OASIS MAX (6 cc/150 mg; Waters Co.) with *N*-vinylpyrrolidone/divinylbenzene hydrophilic–lipophilic balanced copolymer mixed anion-exchange phase. Because MBP and MEHP have carboxyl group and alkyl groups, this anion exchange/reverse phase column was suitable for extracting these metabolites. The SPE cartridge was preconditioned with 15 mL acetonitrile and 5 mL 100 mM ammonium acetate/ammonia solution and then loaded with deconjugated sample in 3 mL 100 mM ammonium acetate/ammonia solution. The SPE cartridge was then rinsed with 5 mL water and 5 mL acetonitrile, after which it was dried under vacuum for 3 min. The sample was eluted with 5 mL acetonitrile containing 1% formic acid. The elution sample solution was dried under a stream of nitrogen at 40°C before resuspension in 300 μL acetonitrile/water (50/50, vol/vol). The final sample solution was analyzed by liquid chromatography-MS/MS.

### Analytical validation by liquid chromatography-MS/MS

Using the liquid chromatography-MS/MS conditions described above, the retention times for MBP and MEHP were 5.6 and 6.4 min, respectively, with a relative SD (RSD) of 0.5–0.7% on 3 different days (*n* = 10). The calibration graphs (peak area ratios of the internal standard versus sample concentration) obtained for MBP and MEHP (slopes 0.014 and 0.017 and intercepts 0.0541 and 0.034, respectively; *r* > 0.999) were linear over the calibration range from 5 ng/mL to 50 μg/mL. The limit of detection (LOD) was 0.5 and 0.6 ng/mL (signal/noise ratio = 3) for MBP and MEHP, respectively. If the observed level was below the lowest calibration standard in the HPLC-MS analysis, we repeated the measurement using a sample volume that was double (or more) that used in the original analysis. The limit of quantitation was 5 ng/mL (signal/noise ratio > 10). The average recovery for MBP and MEHP (10 and 100 ng/mL) in urine samples ranged from 97.8% to 100.8% (RSD < 7.5%, *n* = 6).

### Determination of plasma hormones

Plasma levels of LH, FSH, fT, and E_2_ were measured by radioimmunoassay in a commercial laboratory (SRL Inc., Tokyo, Japan). The reference values for the determinations provided by the laboratory were 1.8–5.2 IU/mL, 2.9–8.2 IU/mL, 14–40 pg/mL, and 20–60 pg/mL, respectively. For LH, FSH, fT, and E_2_, respectively, the LODs were 0.10 mIU/mL, 0.05 mIU/mL, 0.6 pg/mL, and 1.0 pg/mL, and the coefficients of variance (interday variation) were 4%, 3%, 1.6–3.6, and 3–4%. The reference range provided by the commercial laboratory were as follows: LH, 1.8–5.2 mIU/mL; FSH, 2.9–8.2 mIU/mL; fT, 3.3–21.3 pg/mL; and E_2_, 20–59 pg/mL.

### Statistical analyses

We calculated medians, geometric means (GMs), and distribution percentiles of creatinine-adjusted concentrations for urinary levels of MBP and MEHP. Urinary levels of MEHP and MBP and serum levels of FSH, LH, fT, and E_2_ were transformed to log_10_ for statistical analysis. The ratio of LH to fT was calculated by simple division. Values for MEHP and MBP were specified as 0.5 ng/mL and 0.6 ng/mL, respectively, when levels fell below the LOD. GMs were compared between subgroups by the two-sample *t-*test. We estimated daily intake of DEHP for each subject according to the method of Koch et al. (2003a). The standardized partial correlation coefficient was calculated to assess bivariate relationships adjusting for potential confounding variables. We performed a stepwise multiple regression analysis to determine the independent variables [among MEHP, MBP, age, alcohol consumption (yes/no), tobacco smoking (yes/no), body mass index (BMI), SIP, and FSPC] important in predicting the serum concentrations of FSH, LH, fT, and E_2_. The significance levels for entry and inclusion in the model were *p* < 0.05 and *p* < 0.10, respectively. Age was forced into the final model when we assessed the relationship between hormones and the statistically significant variables. Because MBP and MEHP are highly correlated and because the limited sample size in the present study prohibited us from examining the effects of the individual exposure and coexposure, we calculated the total phthalate esters score (TPES) according to the method of Swan et al. (2005). MBP and MEHP were divided into quartiles and assigned values of 0, 1, 2, and 3, respectively. TPES equals the sum of scores for MBP and MEHP. Differences in proportions were tested by the chi-square method or, when the expected values in cells were small, by Fisher’s exact test. A *p*-value < 0.05 (two-tailed) was considered significant.

## Results

The baseline characteristics of all subjects are shown in Table 1. The unexposed workers were comparable in terms of age, marital status, and smoking and alcohol consumption habits, as well as the plastic material exposure index. Subjects in the exposed worker group had worked < 1 year on average, which was significantly lower than the corresponding time for the unexposed workers (*p* < 0.01).

Table 2 shows the GMs and selected percentiles of urinary MBP and MEHP for exposed and unexposed workers. As a reference, the corresponding values of American males from the National Health and Nutrition Examination Survey (NHANES) are also presented (Silva et al. 2004a). MBP and MEHP were detected in all workers, apart from one subject in the unexposed group for which MEHP could not be detected. Exposed workers had significantly higher levels of MBP and MEHP than the unexposed workers. Table 3 shows the concentrations of FSH, LH, fT, and E_2_ for the exposed and unexposed groups. We found significantly lower fT levels in exposed workers than in unexposed workers, but no significant difference between the two groups for FSH, LH, or E_2_.

Table 4 shows the standardized partial correlation coefficients between urinary levels of MBP and MEHP and plasma levels of FSH, LH, fT, E_2,_ and LH/fT in exposed, unexposed, and total workers. MBP and MEHP correlated positively in both the exposed and unexposed groups (Table 4). fT was negatively correlated with MBP and MEHP in the exposed worker group and in all subjects but not in the unexposed group. We found a non-significant negative correlation between FSH and MBP and MEHP in the exposed group (Table 4). There was a positive correlation between FSH and LH only in the unexposed group but not in the exposed worker group. fT levels correlated positively with LH and E_2_ in both the exposed and unexposed groups, and a negative correlation was observed between LH/fT and MBP in the exposed group. We found no associations between MBP, MEHP, LH, and E_2_. A regression analysis showed a significant decrease in fT with increasing TPES (*r* = −0.26, *p* = 0.002).

Table 5 shows the results of multiple regression analyses. A positive correlation was found between both FSH and LH and age, whereas a negative correlation was found between fT and age (*p* < 0.10), MEHP (*p* < 0.01), and alcohol consumption (*p* < 0.10) for all subjects. MBP levels were the only significant predictor of fT levels in the exposed workers. When TPES was included in the model replacing MEHP and MBP, TPES remained as a significant predictor of fT levels for all subjects and exposed workers (data not shown). BMI was the only significant predictor of fT in the unexposed workers. We found a positive correlation between FSPC and E_2_ in the exposed group.

## Discussion

In this cross-sectional study we evaluated the relationship between occupational exposure to DBP and/or DEHP and serum sex hormones in male Chinese workers. Urinary MBP and MEHP were detected in all 137 subjects, except 1 subject in the unexposed group whose urine contained undetectable levels of MEHP. The detection rates of MEHP we found in the present study—100% in exposed workers and 98% in unexposed workers—are higher than the 81% reported for American males by Silva et al. (2004a). By contrast, the detection rate for MBP found here in Chinese workers was similar to that found in American males (Silva et al. 2004a). These findings indicate that exposure to DBP and DEHP is ubiquitous in China and other parts of the world.

The levels of urinary MBP and MEHP in the exposed group (GMs of 644.3 and 565.7 μ/g creatinine, respectively; Table 2) was 5–100 times that of the unexposed group. Of the 74 exposed workers, 3 had urinary MBP levels > 21,000 μg/g creatinine, which is greater than the highest MBP level ever identified in a male patient with ulcerative colitis [16,868 μg/g creatinine (Hauser et al. 2004a)]. The levels for MBP and MEHP were 7.5 and 2.0 times higher in unexposed workers than in American males (Silva et al. 2004a). Because the factory described in the present study used waste plastic materials as raw materials, it is possible that more DBP and/or DEHP were used than in other PVC flooring factories. Poor environmental control of dust and vapor and the insufficient use of air masks are likely contributors to high DBP and DEHP exposure. In the present study, workers handled raw and mixed materials containing DBP and/or DEHP on production lines, with dermal contact occurring frequently and directly via hands, arms, and other parts of the body, or indirectly through contaminated work clothes. The high temperature attained in the workshops may have increased the levels of DEHP and DBP in the air as well as the dermal absorption rate. It is also possible that workers ingested DBP and/or DEHP by drinking or eating contaminated water and food. We suspect that the high levels of urinary MBP and MEHP in the exposed worker group are caused primarily by air pollution and heavy dermal contamination.

The levels of urinary MBP and MEHP among the unexposed workers (median 113.5 and 5.4 μg/g creatinine, respectively; Table 2) were similar to those found among German males [111.0 and 8.2 μg/g creatinine, respectively (Koch et al. 2003b)], and 7 to 2 times those found among American males (Silva et al. 2004a). A number of studies have demonstrated widespread pollution of DBP and DEHP in environmental and biological samples in China (Hu et al. 2003; Zhang et al. 2003). Before the present study, there were no reports of urinary MBP or MEHP levels in China. The significant correlation between urinary MBP and MEHP in the exposed (*r* = 0.72, *p* < 0.001) and unexposed workers (*r* = 0.51, *p* < 0.001) suggests that exposure to DBP and DEHP in working environments and in the general environment is simultaneous. A similar correlation between MBP and MEHP (*r* = 0.37, *p* < 0.0001) was reported among American males by Silva et al. (2004a).

The median of estimated daily intake (DI) of DEHP in the exposed group was 48.2 μg/kg body weight/day, which was significantly higher than that of the unexposed group (0.5 μg/kg body weight/day). Of 74 exposed workers, 30 (32.1%) had a DI above the tolerable daily intake (TDI; 37.0 μg/kg body weight/day) (Committee for Toxicity, Ecotoxicity and the Environment 1998).

In the present study we demonstrate for the first time a significant negative correlation between serum fT and urinary MBP (*r* = −0.24, *p* = 0.006) and MEHP (*r* = −0.24, *p* = 0.005) (Table 4). Furthermore, the exposed workers had significantly lower fT levels than the unexposed workers (8.4 vs. 9.7 μ/g creatinine, *p* = 0.019; Table 3). Multiple regression analyses showed that age, MEHP, and alcohol consumption were significantly related to reduced levels of fT. MBP was the only significant predictor of fT in the exposed workers. When TPES, MBP, and MEHP were assessed in the same model, only TPES was retained in the model for total subjects and exposed workers, indicating that coexposure to both MBP and MEHP was responsible for the reduction in fT. The negative relationship we observed between fT, age, and alcohol consumption is consistent with findings from other studies (Emanuele and Emanuele 1998; Ma and Zheng 2004).

Toxicologic studies have invariably shown that MEHP and MBP are toxicants of LCs and SCs in the testis. The MEHP-induced inhibition of testosterone production in LCs is thought to be associated with decreased pituitary LH secretion and reduced steroidogenic enzyme activity (Akingbemi et al. 2001). Maternal exposure to DBP leads to a decrease in testosterone biosynthesis by reducing cholesterol synthesis, transport, and storage in fetal LCs (Barlow et al. 2003). The relationship between testosterone and PE exposure has been explored in a few epidemiologic studies in recent years. Yin et al. (1998) reported a non-significant decrease of serum fT levels in 85 male Chinese workers with occupational exposure to di-octyl phthalate compared with 72 controls (30.7± 2.0 nmol/L vs. 36.0 ± 3.3 nmol/L). Zhang et al. (2003) reported a nonsignificant negative correlation between serum fT and serum DEP (*r* = −0.36, *p* = 0.398) and DBP (*r* = −0.23, *p* = 0.588) in 8 healthy Chinese males. Duty et al. (2005) observed a negative correlation between MEHP and testosterone (*r* = −0.17, *p* < 0.05) in 295 American males. Main et al. (2006) reported that MBP was negatively correlated with fT (*r* = −0.22, *p* = 0.033) in 96 3-month-old boys. Jonsson et al. (2005) examined the relationships between various urinary PEs and gonadal hormones among Swedish males, but they did not observe any significant effects of PEs on serum fT. In a study of 19 adolescents exposed to DEHP as neonates under extra-corporeal membrane oxygenation support, levels of FSH, LH, fT, and E_2_ were within the normal range (Rais-Bahrami et al. 2004). The first evidence that prenatal PE exposure can adversely affect human male reproductive development was provided by Swan et al. (2005). Although reduced testosterone production in LCs was put forward as a likely cause of adverse male reproductive development, the authors did not measure either testosterone or other gonadal hormones. It should be noted that testicular atrophy and decreased sperm production observed in rats occurs at PE exposure levels substantially higher than human exposure levels in the general environment. To clarify the effects of PE exposure on human sex hormones, more studies are warranted in populations with various exposure levels, especially populations with high exposure (Mantovani et al. 1999).

The low level of fT observed in workers exposed to PEs is mostly due to the exposure to DBP and DEHP in the working environment. In the present study, MBP and MEHP levels in the exposed workers were 5–100 times the levels observed in the unexposed workers and may be in the range required to induce human testicular toxicity. The results from the present study support the notion from animal studies that PEs can suppress testosterone biosynthesis in humans.

The mechanisms underlying testicular toxicity that lead to adverse reproductive effects are complex. Normally, when androgen biosynthesis is sufficiently depressed, the lowered serum fT concentrations act via negative feedback mechanisms to induce increased LH output from the pituitary gland. LH then stimulates LCs to secrete more testosterone, which in the course of time, and acting via the same pathway, restores pituitary LH secretion to normal levels. In this manner, the negative feedback mechanism serves as a homeostatic control for the HPT axis (Kumar 2004). Compared with the unexposed workers, exposed workers had nonsignificant reductions of FSH and LH levels. The decreased LH concentration was not consistent with our hypothesis that LH should increase in response to reduced fT levels. The positive correlation between LH/fT and MBP (*r* = 0.216, *p* = 0.034) and MEHP (*r* = 0.146, *p* = 0.110) in the exposed workers was caused by a decrease in fT, not by an increase in LH. The most likely explanation for the simultaneous occurrence of significantly decreased fT and nonsignificant decreases of LH and FSH levels in the exposed worker group is that the combined exposure to high levels of MBP and MEHP may have caused dysfunction of both testosterone biosynthesis in the testis and the normal feedback regulation of the HPT axis. The negative relationship between fT and MBP/MEHP in the exposed group was not present in the unexposed group, a finding consistent with the results of a study of Swedish males (Jonsson et al. 2005). This implies that the relatively low level of environmental PE exposure may not cause significant serum fT reduction or that the reduction was subtle and compensated by the feedback regulation of the HPT axis. Other raw materials used in the production line of this factory, including PVC resin, azodicarbonamide as a forming agent, and calcium carbonate as a filler, would not have contributed to the decreased fT. FSH, LH, and testosterone play crucial roles in the initiation, maintenance, and restoration of spermatogenesis. Toxicants that damage the LCs can lead to a reduction in the secretion of testosterone, which in turn can affect SC function and spermatogenesis. Although spermatogenesis may be maintained by intratesticular testosterone produced in response to LH stimulation of the LCs, it is generally recognized that combined stimulation with FSH and LH leads to maximal sperm production. The circulating concentration of FSH is thought to provide the signal that sets the level of sperm production above the basal rate induced by intratesticular testosterone (Plant and Marshall 2001). The simultaneously decreased levels of FSH, LH, and fT among exposed workers may have an adverse effect on spermatogenesis. Although the normal range of serum fT in Chinese males is 5.6–10.2 ng/dL (Liao and Cao 2001), in the present study we found that 9.5% (7/74) of exposed workers had serum fT levels < 5.6 ng/dL, which was not significantly higher than the 4.8% (3/63) we observed in the unexposed group. Understanding the clinical relevance of the decreased serum fT levels requires assessment of sperm levels. In the present study, we found no obvious effects of daily plastic material usage on either PE levels or serum hormones.

The results of the present study must be interpreted with caution because the phthalate and hormone levels were determined from single spot urine and blood samples. It is well known that there is significant minute-to-minute variation in endogenous serum LH and FSH concentrations. For this reason, it is possible that spot sampling may cause a bias by not reflecting average hormone levels. Phthalates have short half-lives, and urinary samples reflect only recent exposure. In support of the methodology we used in the present study, Hauser et al. (2004b) reported that a single urine sample was moderately predictive of each subject’s exposure over a 3-month period. Because the subjects in the exposed group had worked in the factory an average of < 1 year, the low levels of fT would have been caused by the current exposure to high levels of PEs. To further evaluate the effect of PEs on reproductive function, other potentially important biomarkers such as other PEs (e.g., monoethyl phthalate), inhibin B, and gonadotropin-releasing hormone could be assessed in addition to measuring sperm levels.

## Conclusions

In the present study we observed a modest and significant reduction of serum fT in workers with higher levels of urinary MBP and MEHP compared with unexposed workers. fT was significantly and negatively correlated with urinary levels of DBP and DEHP. In future studies, analysis of the effects of PE exposure on gonadotropin and steroid hormone levels should form part of an overall risk assessment for PEs.

## Correction

In the the description of the MS/MS procedure for MEHP and MEHP-d_4_ in “Materials and Methods” of the article published online, the molecular transitions used in multiple reaction monitoring were incorrect; they have been corrected here.

## Figures and Tables

**Table 1:** Demographic characteristics [no. (%)] of exposed (*n* = 74), unexposed (*n* = 63), and total workers (*n* = 137).

Characteristic	Exposed	Unexposed	All
Age at interview (years)^a^	33.5 ± 9.4	34.3 ± 9.9	33.9 ± 9.6
Age (years)			
< 20	5 (7)	4 (6)	9 (7)
20–29	25 (34)	18 (29)	43 (31)
30–39	21 (28)	20 (32)	41 (30)
40–49	20 (27)	17 (27)	37 (27)
≥50	3 (4)	4 (6)	7 (5)
Years working in current job^a^	1.0 ± 0.8*	2.6 ± 5.5	1.7 ± 3.8
Marriage status			
Single	21 (28)	15 (24)	36 (26)
Married	50 (68)	48 (76)	98 (72)
Divorced	3 (4)	0	3 (2)
Smoker	44 (60)	39 (62)	83 (61)
Drinker	40 (54)	40 (64)	80 (58)
SIP score			
0	33 (45)	24 (38)	57 (42)
1	27 (36)	29 (46)	56 (41)
2	10 (14)	8 (13)	18 (13)
3	4 (5)	2 (3)	6 (4)

a Mean ± SD.

* *p* < 0.01.

**Table 2:** Selected percentiles and GMs of urinary MBP and MEHP concentrations (μg/g creatinine) among exposed workers, unexposed workers, and the American male population.

		Percentiles							
Marker/subjects	No. (%)^a^	10th	25th	50th	75^th^	90th	95th	GM	*p*-Value
MBP									
Exposed	74 (100)	156.7	252.1	548.4	1492.6	2455.5	8781.2	644.3	
Unexposed	63 (100)	58.2	74.7	113.5	206.8	338.2	434.5	129.6	< 0.001
NHANES^b^	1,215 (99)	6.5	10.2	17.0	28.6	49.1	63.6	17.3	
MEHP									
Exposed	74 (100)	78.0	209.6	562.3	1884.4	3303.7	5379.7	565.7	
Unexposed	63 (98)	2.0	3.7	5.4	9.9	15.4	23.2	5.7	< 0.001
NHANES^b^	1,215 (81)	< LOD	1.3	2.8	5.6	10.3	21.6	2.9	

a Sample size and percentage of detection.

b Data from Silva et al. (2004a).

**Table 3:** Concentrations (mean ± SD) of log_10_-transformed serum FSH, LH, fT, and E_2_ among exposed (*n* = 74) and unexposed (*n* = 63) workers.

Hormone	Exposed	Unexposed	*p*-Value
FSH	5.0 ± 1.5	5.4 ± 1.7	0.360
LH	4.3 ± 1.5	4.9 ± 1.7	0.102
T	8.4 ± 1.5	9.7 ± 1.4	0.019
E_2_	22.4 ± 1.6	20 ± 1.7	0.187

**Table 4:** Standardized partial correlation coefficients between levels of urinary metabolites and plasma hormones in all subjects.

	Exposed^a^						Unexposed^a^						All^a^					
	MEHP	FSH	LH	fT	E_2_	LH/fT	MEHP	FSH	LH	fT	E_2_	LH/fT	MEHP	FSH	LH	fT	E_2_	LH/fT
MBP^b^	0.716	−0.180	0.087	−0.253	−0.029	0.216	0.549	0.002	0.078	0.095	−0.061	−0.032	0.799	−0.103	−0.042	−0.237	0.032	0.073
*p*-Value	0.000	0.129	0.466	0.032	0.808	0.034	0.000	0.998	0.550	0.467	0.639	0.402	0.000	0.107	0.632	0.006	0.712	0.199
MEHP^b^		−0.191	0.035	−0.198	0.007	0.146		0.092	0.127	−0.045	−0.128	0.109		−0.103	−0.109	−0.242	0.077	0.035
*p*-Value		0.109	0.768	0.095	0.955	0.110		0.480	0.330	0.728	0.325	0.202		0.235	0.207	0.005	0.376	0.345
FSH^b^			0.156	0.084	0.051	0.093			0.319	−0.081	−0.229	0.328			0.262	0.032	−0.075	0.224
*p*-Value			0.192	0.486	0.669	0.218			0.012	0.533	0.076	0.005			0.002	0.713	0.385	0.004
LH^b^				0.315	0.229	0.715				0.225	0.167	0.855				0.294	0.177	0.784
*p*-Value				0.007	0.053	0.000				0.082	0.199	0.000				0.001	0.040	0.000
fT^b^					0.465	−0.402					0.418	−0.255					0.402	−0.318
*p*-Value					0.000	0.000					0.001	0.023					0.000	0.000
E_2_^b^						−0.101						−0.022						−0.058
*p*-Value						0.200						0.432						0.251

a Standardized partial correlation coefficients were adjusted for age and alcohol consumption status (yes/no).

b Log_10_-transformed MBP, LH, FSH, LH, fT, and E_2_.

**Table 5:** Multiple regression analyses predicting levels of FSH, LH, fT, and LH in exposed, unexposed, and all workers.

	FSH^a^			LH^a^			fT^a^			E_2_^a^		
	Exposed	Unexposed	All	Exposed	Unexposed	All	Exposed	Unexposed	All	Exposed	Unexposed	All
Age^b^	0.257*	0.585^#^	0.474^#^	0.305^#^	0.450^#^	0.383^#^	−0.097	−0.129	−0.152*	−0.159	−0.069	−0.138
BMI	—	—	—	—	—	—		0.287**	—	—	—	—
MBP	—	—	—	—	—	—	−0.257**	—	—	—	—	—
MEHP	—	—	—	—	—	—		—	−0.235^#^	—	—	—
Drinking alcohol	—	—	—	—	—	—	—	—	−0.230*	—	—	—
FSPC	—	—	—	—	—	—	—	—	—	0.330^#^	—	—
Adjusted *R*^2^	0.159	0.331	0.219	0.081	0.19	0.14	0.058	0.084	0.098	0.012	0.09	0.019
*p*-Value	0.001	< 0.001	< 0.001	0.008	< 0.001	< 0.001	0.045	0.043	0.001	0.177	0.022	0.109

—, Excluded from model.

a Values are beta coefficients except for *R*^2^ and *p-*value.

b Age was forced in each model.

* *p* < 0.10.

** *p* < 0.05.

# *p* < 0.01.
